# Bone marrow donor selection and characterization of MSCs is critical for pre-clinical and clinical cell dose production

**DOI:** 10.1186/s12967-019-1877-4

**Published:** 2019-04-17

**Authors:** Alpa Trivedi, Byron Miyazawa, Stuart Gibb, Kristen Valanoski, Lindsay Vivona, Maximillian Lin, Daniel Potter, Mars Stone, Philip J. Norris, James Murphy, Sawyer Smith, Martin Schreiber, Shibani Pati

**Affiliations:** 1 Department of Laboratory Medicine, University of California, San Francisco, 513 Parnassus Avenue, HSE 760, San Francisco, CA 94143 USA; 2 Vitalant Research Institute, University of California, San Francisco, San Francisco, USA; 3 Department of Trauma, Surgical Critical Care, and Acute Care Surgery, Oregon Health and Science University, Portland, USA

**Keywords:** Stem cell therapy, Bioprocessing, Viability, Differentiation, Donor variability

## Abstract

**Background:**

Cell based therapies, such as bone marrow derived mesenchymal stem cells (BM-MSCs; also known as mesenchymal stromal cells), are currently under investigation for a number of disease applications. The current challenge facing the field is maintaining the consistency and quality of cells especially for cell dose production for pre-clinical testing and clinical trials. Here we determine how BM-donor variability and thus the derived MSCs factor into selection of the optimal primary cell lineage for cell production and testing in a pre-clinical swine model of trauma induced acute respiratory distress syndrome.

**Methods:**

We harvested bone marrow and generated three different primary BM-MSCs from Yorkshire swine. Cells from these three donors were characterized based on (a) phenotype (morphology, differentiation capacity and flow cytometry), (b) in vitro growth kinetics and metabolic activity, and (c) functional analysis based on inhibition of lung endothelial cell permeability.

**Results:**

Cells from each swine donor exhibited varied morphology, growth rate, and doubling times. All expressed the same magnitude of standard MSC cell surface markers by flow cytometry and had similar differentiation potential. Metabolic activity and growth potential at each of the passages varied between the three primary cell cultures. More importantly, the functional potency of the MSCs on inhibition of endothelial permeability was also cell donor dependent.

**Conclusion:**

This study suggests that for production of MSCs for cell-based therapy, it is imperative to examine donor variability and characterize derived MSCs for marker expression, growth and differentiation characteristics and testing potency in application dependent assays prior to selection of the optimal cell lineage for large scale expansion and dose production.

**Electronic supplementary material:**

The online version of this article (10.1186/s12967-019-1877-4) contains supplementary material, which is available to authorized users.

## Background

The field of regenerative medicine and cellular therapies is an expanding area of development and exploration in challenging diseases with few therapeutic options. Cell based therapies are gaining momentum for treatment of diseases such as stroke, trauma, diabetes, and cancer [1]. Mesenchymal stem cells (MSCs; a.k.a mesenchymal stromal cells) have been tested extensively for both autologous and non-autologous therapies and are utilized in the fields of regenerative medicine, tissue engineering and immunomodulation.

MSCs were first discovered by Friedenstein and colleagues in the 1960s as a component of bone marrow (BM) derived cells that adhere to plastic and have a typical spindle shaped morphology [2]. They also express surface markers such as CD44, CD90 and CD105 and exhibit “trilineage” (adipogenic, osteogenic and chondrogenic) differentiation potential [3]. In addition to their capacity to differentiate into adipocytes, osteocytes or chondrocytes, and regenerate into particular tissue types, MSCs possess potent anti-inflammatory, vasculo-protective and immunomodulatory effects primarily mediated through the soluble factors and extracellular vesicles (EVs) they release in their secretome [4].

There are over 600 clinical trials listed for MSCs, primarily in Phase 1, 2 and 3 trials in the treatment of graft vs. host disease [4]. Clinical efficacy in multiple trials with BM-MSCs has been variable, with results that are not commensurate with pre-clinical findings in vitro and in vivo in animal models of disease. One of the reasons for this could be the quality of the MSC cell product being tested [5]. Donor to donor variability in phenotype and growth kinetics causes significant inter-individual heterogeneity in the cell product [6], which potentially results in inconsistent outcomes in preclinical to clinical translation in patients [4]. Hence it is critical in the cell production process of cell-based therapies that potential donors are screened and that cells from each individual donor are characterized for their differentiation potential, growth kinetics, storage conditions and potency in the indicated disease application.

In this paper, we focus specifically on the production of a MSC cell product for downstream testing in a pre-clinical swine model of polytrauma induced acute respiratory distress syndrome (ARDS). The swine model is characterized by histopathological lung injury, inflammation and decreased PaO_2_/FiO_2_ ratio (partial pressure of arterial oxygen (PaO_2_) to percentage of inspired oxygen (FiO_2_) ratio, which measures oxygenation level of blood), all characteristic of clinical ARDS and potential targets for MSC therapy [7]. We have chosen to test swine MSCs in the swine model, rather than human MSCs, for potential xenocompatibility issues. Swine MSCs have been reported to have similar biological activities to human MSCs [8, 9].

In this paper, we describe our endeavors to produce therapeutic swine MSC doses on a large scale using a donor with the optimal potency and growth kinetics suitable for large scale expansion. We aimed to characterize three swine donors and subsequently selected one donor for dose production and testing in vivo. MSCs from three different donor pigs were isolated and cultured under identical conditions and characterized at an early passage for marker phenotype, morphological phenotype, proliferation capacity, differentiation potential, and functional assays of potency. The swine MSCs were grown utilizing a bioreactor system that offers large scale automated cell expansion in a closed loop process. Our findings suggest that there is donor and cell variability in the MSCs derived from the BM of the three swine. Based on our assays, we were able to identify an optimal donor from which all pre-clinical doses for the in vivo swine studies will be produced.

## Materials and methods

### Bone marrow harvest

All experiments were approved by the OHSU Institutional Animal Care and Use Committee (IACUC) and the Department of Defense Animal Care and Use Review Office (ACURO). All experiments also adhered to the National Institutes of Health Guide for the Care and Use of Laboratory Animals. Six-month old female Yorkshire swine (Oak Hill Farms, Glen Ellen, CA) weighing between 40 and 50 kg were anesthetized using tiletamine (8 mg/kg IM) and glycopyrrolate (0.01 mg/kg IM). A 6f bone marrow biopsy needle was inserted into the anterior pelvis and a single collection of 50–100 ml of bone marrow was aspirated into a heparin-containing syringe. The bone marrow was then transferred into a conical tube, packaged with ice packs, and then shipped via over-night shipping to UCSF. The amount and overall quality of bone marrow aspirate was similar between the donors tested.

### Isolation of MSCs from bone marrow

**Fig. 1:**
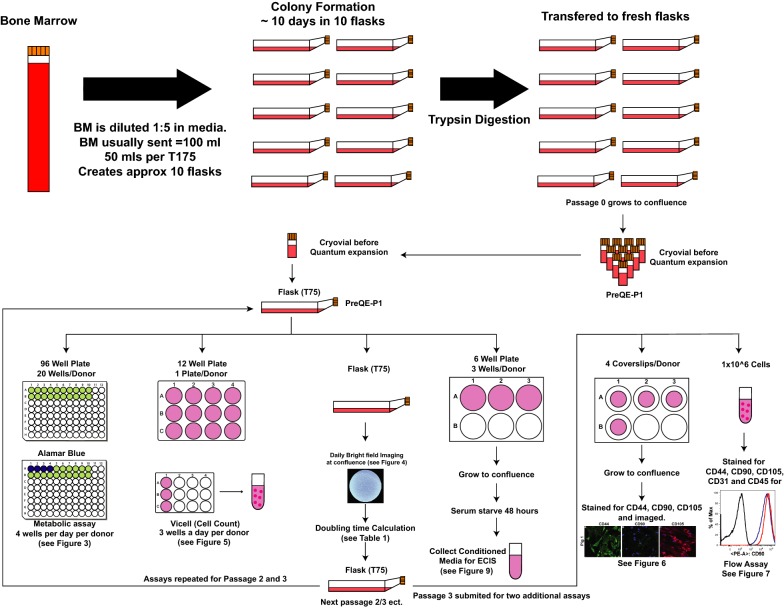
Schema illustrating procedural steps to generate MSCs and expansion in flasks to characterize cells. Heparinized bone marrow aspirate was delivered within 24 h of extraction, diluted and seeded in tissue culture flasks using MSC growth media. Following initial wash steps colony formation occurred (P0 generation). To provide a homogenous population for bioreactor expansion, cells were mixed, replated and grown to sub confluence. This was termed as pre-quantum expansion passage 1 (PreQE-P1). These cells were expanded manually and assayed for metabolic activity, cell proliferation, morphology, cell marker expression, and potency

Bone marrow aspirate was filtered with MACS Smart Strainers (pore size 100 µm) to remove any coagulated matter. Loss by volume was typically less than 5%. Filtered bone-marrow aspirate was then diluted 1:5 with media known to promote growth of MSCs [minimal essential medium (MEM) alpha, 10% MSC-grade fetal bovine serum (FBS), Glutamax, 10 µg/ml Gentamicin]. FBS is USDA certified and has been tested and qualified to support clonal efficiency of MSC derived from bone marrow and support cell expansion and differentiation. After 2 days of incubation at 37 °C, 5% CO_2_, media was removed, and adherent cells underwent 5 washes with PBS. Removal of red blood cells was confirmed by microscopy. Colonies of MSCs were then allowed to grow for a further 5 days. Following 7 days of growth in the primary tissue culture flasks, cells were trypsinized and were re-plated to form a homogenous population of cells. Seven days was selected as a time point for the first trypsinization, as this is sufficient to expand a significant number of cells without the colonies touching/overlapping. We generally observed a cell confluence between 60 and 70%. Furthermore, this time point was selected to prevent any emergence of senescence with smaller colonies. We did not observe any significant/unusual growth rates in the cells derived from the 3 pigs at this time point. The generation of colonies generated after the first trypsinization was designated Passage Zero (P0). When cells in these flasks reached confluence, they were trypsinized and frozen in cryovials for expansion in quantum. These cells were designated as pre-quantum expansion passage 1 (PreQE-P1) (Fig. 1).

### Generation of conditioned medium

**Fig. 2:**
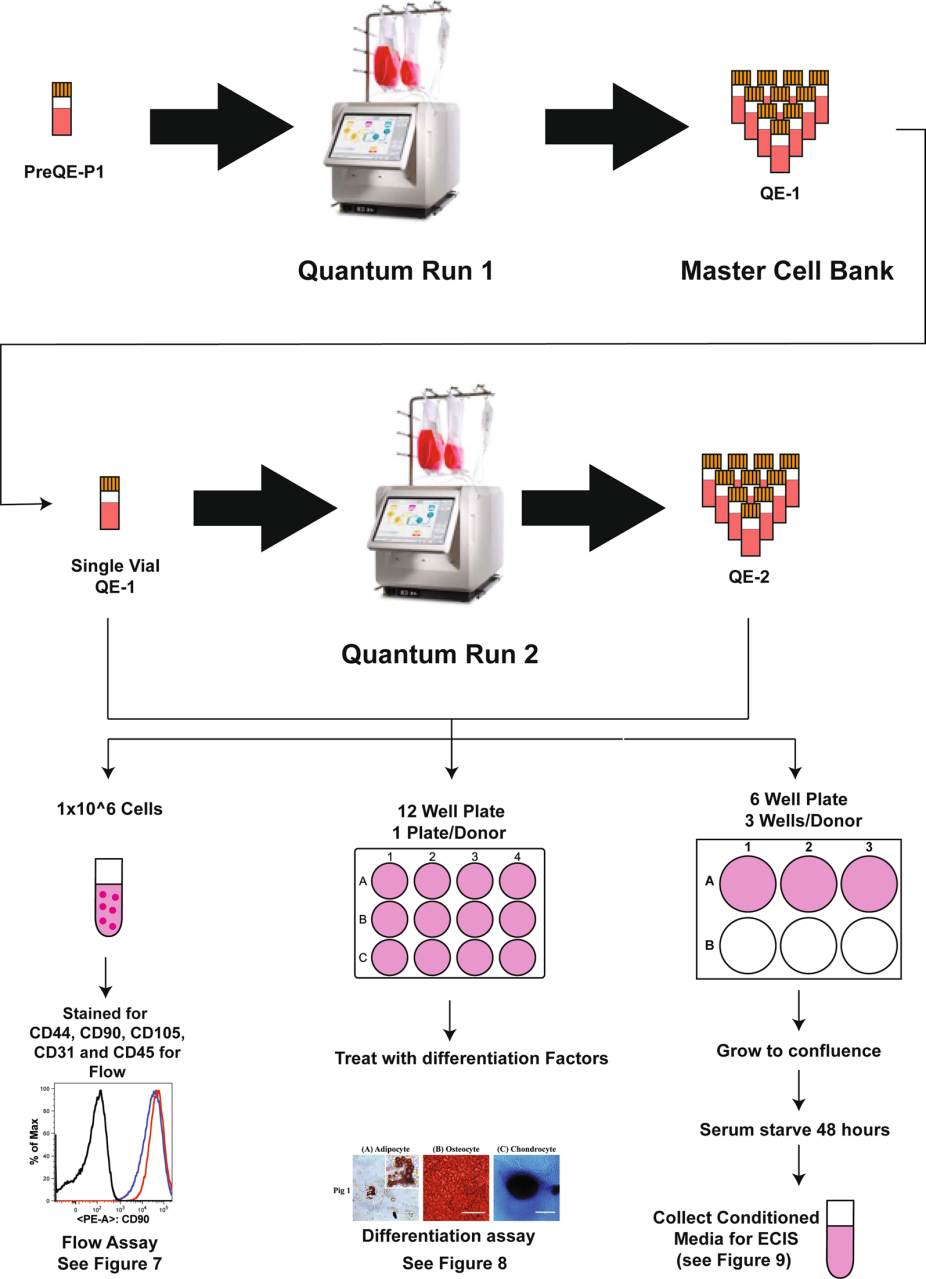
Schema illustrating procedural steps to generate therapeutic MSC doses. Pre-quantum expansion passage 1 (PreQE-P1) cells from Pigs 1 and 3 were seeded onto Quantum bioreactor. A single quantum run then yielded the cell bank quantum expansion 1 (QE-1) from which additional noncommittal quantum expansions generated the therapeutic doses termed as quantum expansion 2 (QE-2). During quantum expansion, cells were tested for metabolic activity and cells from QE-2 were then tested in in vitro assays of marker expression, differentiation and potency

To compare cells from all 3 donors in in vitro assays, we propagated the cells manually in tissue culture flasks for all of the following experiments. This will also avoid the bias of different growth conditions that may affect cell characteristics. Cells from all three donors were expanded manually in flasks up to passage 3 and plated for different assays at each of the passages (see Fig. 1). To generate conditioned medium, cells were seeded at a density of 250,000 cells per well in a 6 well plate and allowed to grow to confluence in complete MSC medium (3 wells/cell type/passage). Similarly, cells from Pigs 1 and 3 that were expanded on quantum (QE-2) (Fig. 2) were seeded at a density of 250,000 cells per well in a 6 well plate and allowed to grow to confluence in complete MSC medium. Once the cells reached confluence, they were serum starved for 48 h and medium was collected, centrifuged to remove dead cell debris and stored at − 80 °C until evaluated in functional assays. Serum starvation is standard and is done so that the effects of what is secreted by the cell in the culture medium is not confounded by the factors present in the serum. Secondly, serum starvation is also a stress response under which cells secrete all factors along with exosomes and microvesicles.

### In vitro cell analysis

#### Cell metabolic assay

Mesenchymal stem cells (MSCs) at PreQE-P1, and manually expanded passages 2 and 3 from all three donors were propagated in Minimum Essential Medium (Alpha MEM, ThermoFisher) with 10% fetal bovine serum (ThermoFisher) and 0.1% gentamicin (ThermoFisher). For metabolic assay, MSCs starting at each of the individual passages were seeded at a density of 12,000 cells per well (4 wells were assayed/day/donor, for a total of 5 days assay time) in a 96-well plate (ThermoFisher) (Fig. 1). In addition, media alone served as negative no cell control for each day (4 wells per day). Metabolic activity was analyzed using the Alamar blue assay following the manufacturer’s protocol (Bio-Rad). Briefly, first assay was performed at 4 h after initial seeding, Alamar blue dye was added to wells to be analyzed at this time point along with wells that served as negative control, at 1× concentration. Rest of the plated wells were left untouched and were analyzed over the course of the next 4 days. After a 4 h incubation with dye, the absorbance was read on plate reader (Epoch, Biotek) at 570 nm and 600 nm. All resulting values were adjusted for background by comparison to no-dye control wells. Wells without cells were utilized to determine a correction factor. Percentage consumption of dye was calculated by comparing the 570 nm value to the corrected 600 nm. This assay was repeated every 24 h using different wells/day.

#### Cell morphology

Cells from all three donors seeded from the three passages (PreQE-P1, manually expanded passages 2 and 3) were imaged on a phase contrast microscope using a 20× objective (ECHO, revolve) (Fig. 1).

#### Cell proliferation assay

Manually expanded MSCs from three donors from PreQE-P1, passage 2 or passage 3 were seeded at 50,000 cells per well in 12-well plates (3 wells/day/donor) (Fig. 1). At 24 h after plating, cells from each donor were counted in triplicate using a Vi-Cell XR viability analyzer (Beckman Coulter). Parameters set were as follows, 15–30 microns diameter, with a minimum circularity of 0, a “low” decluster degree, a cell sharpness of 100, and a cell brightness of 85%. Cells were determined to be viable if their cell spot area was 10% of the total cell and if their cell spot brightness was 75%. This assay was repeated for 4 days in a 12 well plate format with different wells trypsinized daily and used for cell count and viability determination. Known number of cells were also plated on T75 flask from which cells were also harvested after 1 week of plating (Fig. 1) and doubling time and fold increase were evaluated as follows.

$${\text{Population}}\;{\text{doubling}} = \frac{1}{\log \left( 2 \right)} \times \log \left( {\frac{Cx\left( t \right)}{Cx\left( 0 \right)}} \right)$$

where Cx(t) and Cx(0) are the cell numbers at the end and start of exponential growth phase respectively; t is the time (h)

$${\text{Fold}}\;{\text{increase}} = Cx\left( f \right)/Cx\left( 0 \right)$$

where Cx(f) is the final cell number at the end of passage and Cx(0) is the initial number of cells plated.

### Surface marker expression

In addition, cells at passage 3 were immunophenotyped (Fig. 1) and stained for CD44 (Stem Cell Technologies), CD90 (Stem Cell Technologies), and CD105 (Novusbio). Primary antibodies were known to cross react with pig antigen and were detected with fluorescently tagged secondary antibodies, Alexafluor 488 anti-rat IgG (Life Technologies), Alexafluor 488 anti-mouse IgG (Life Technologies), and Alexafluor 594 anti-mouse Fab fragment (Cell Signaling). Images were captured using a Nikon 80i Epifluorescence microscope (Nikon) and a RTcmos camera (SPOT Imaging).

### Expanding cells on the quantum bioreactor platform

To generate therapeutic MSC doses, cells underwent two cell expansions on the quantum bioreactor platform (Fig. 2). PreQE-P1, available in limited supply from the isolation and expansion from bone-marrow aspirate were expanded to form a quantum expansion 1 (QE-1) generation which formed the ‘MSC cell bank’, from which aliquots would be used for subsequent expansions on quantum [quantum expansion 2 (QE-2)]. This generation will be used as the cellular therapeutic for in vivo model and also for cell potency characterization. Only cells from pigs 1 and 3 were expanded on quantum and tested head to head. Procedurally both the QE-1 and QE-2 expansions on the quantum bioreactor platform were the same. In brief, prior to introducing cells into the quantum bioreactor a disposable cell expansion set was loaded onto the machine. The disposable set was primed with Ca^2+^/Mg^2+^ free phosphate buffered saline (PBS; Life Technologies) to purge sterile air from the set and the set was then coated overnight with 5 mg fibronectin (Corning). Fifteen million cells were loaded onto the Quantum and allowed to attach overnight. A programmed feed rate protocol was then initiated. Base media was Minimal Essential Medium (MEM) alpha supplemented with 10% MSC grade FBS and Glutamax (ThermoFisher). Feed rates were ramped every 24 h by the computer program, starting at 0.1 ml/min, culminating at 1.2 ml/min. As cells cannot be directly observed during closed system expansion in the quantum, the progression of cell expansion was monitored through glucose and lactate levels in culture media sterile sampled daily in triplicate using hand held devices (Contour and Nova Biomedical respectively). Post-expansion QE-1 cells forming the cell bank were resuspended in Cryostor 10 (BioLife solutions) at 5 million cells/ml, transferred to 2 ml pre-chilled cryovials (NUNC) and control rate frozen to − 80 °C in CoolCell LX alcohol free controlled rate freezing units (Biocision) before transfer to LN_2_ for long term storage. Post-expansion QE-2 cells forming the therapeutic doses were similarly cryopreserved at a density of 10 million cells/ml in 5 ml cryovials, with a total 50 million cells/vial. Cells generated from Quantum at passage QE-2 were used to generate conditioned medium to be tested in ECIS, cell marker expression by flow cytometry and cell differentiation potential.

### Flow cytometry

Cryopreserved MSCs from QE-1 and QE-2 for Pigs 1 and 3 and passage 3 cells (manually expanded) for Pig 2 were thawed at 37 **°**C, counted with a hemocytometer, and 1 × 10^6^ cells were transferred to polystyrene tubes for incubation with staining buffer [DPBS supplemented with 0.2% BSA (BD Biosciences)] and the appropriate antibody cocktail for 30 min at room temperature. CD44-APC, CD90-PE (Stem Cell Technologies), and CD105-Alexa405 (Novus Biologicals) were used to identify the MSC populations. CD31-FITC, CD45-FITC, and Swine-Leukocyte-Antigen Class II-FITC (SLA-DRII) (GeneTex) were used to determine the presence of non-MSC lineage positive cells in the expanded MSCs. Prior to acquisition on the BD LSRII flow cytometer, cells were stained for 10 min using 7AAD viability dye (BD Biosciences). Data were analyzed using FlowJo software version 9.9 (FlowJo). A compensation matrix was generated using single color stains on MSCs. Doublets were excluded using FSA vs. FSH gating and debri was excluded using FSA vs. SSC gating for all samples before gating on total live cells negative for 7AAD (BD Biosciences). ‘Fluorescence Minus One (FMO)’ controls were used to identify positive populations.

### Tri-lineage differentiation of MSCs

Cryopreserved MSCs from QE-2 of donor Pigs 1 and 3 were thawed at 37 °C and plated in MSC growth medium [MEM Alpha 1X (ThermoFisher)] supplemented with 10% FBS and 5 µg/ml gentamicin (ThermoFisher) and incubated overnight at 37 °C before splitting into three cultures for each lineage. Adherent cells were detached with 0.25% Trypsin (ThermoFisher). For adipocyte and osteocyte cultures, 2 × 10^5^ MSCs were grown overnight in a 12 well plate containing MSC growth medium before switching to appropriate differentiation medium (Adipogenesis differentiation kit, Osteogenesis differentiation kit; ThermoFisher). For chondrocyte cultures, 5 µl droplets at a concentration of 1 × 10^7^ cells/ml were placed into a 37 °C CO_2_ incubator for 2 h in a 96 well U-bottom plate to allow for spheroid formation before adding 100 µl of differentiation medium (Chondrogenesis differentiation kit; ThermoFisher). All cultures were fed with fresh pre-warmed differentiation media every 2–3 days. After 5–7 days, adipocytes were stained with Oil Red O (Sigma Aldrich) for 20 min. After 14–21 days, osteocytes were stained with 2% Alizarin Red S (Sigma Aldrich) for 5 min. After 14–21 days, chondrocytes were stained with 1% Alcian Blue for 30 min. Prior to staining, all cells were fixed with Image-IT fixative solution containing 4% formaldehyde (ThermoFisher). Bright field images were captured throughout culture and following each stain (EVOS digital inverted microscope, Life Technologies and Leica CTR6500, JH Technologies).

### Transendothelial electrical resistance

The barrier integrity of human pulmonary microvascular endothelial cells (HPMVEC, Promocell) monolayers was measured using an electric cell-substrate impedance sensing system (ECIS 1600, Applied BioPhysics). An incline or decline in transendothelial electrical resistance (TEER) across the cell monolayers, indicated decreased or increased endothelial paracellular permeability respectively. HPMVECs were grown on l-cysteine-reduced, 96-well plates containing electrodes in each well. Cells were treated with 50% plain MSC growth media as a control, or a dose curve of conditioned media generated from the different MSC donors (passage 3 from manually expanded cells for all 3 cell donors or QE-2 cells for Pigs 1 and 3), for 1 h, and then challenged with thrombin (Sigma-Aldrich) at a concentration of 0.2 U/ml. Monolayer resistance at 4/16/64 kHz was analyzed in 8-min intervals. Data were normalized to the mean resistance of cell monolayers before the treatments. Resulting resistance values were calculated in comparison to control samples as an area under curve (AUC), or as a maximum decrease in resistance.

### Statistics

Statistical analyses were conducted using GraphPad Prism 7.0 (GraphPad Software). Metabolic assay and proliferation analysis were compared between donors using two-way analysis of variance (ANOVA) for tests between subject and within subject effects, followed by Sidak’s multiple comparison test. TEER values were compared to control samples using one-way ANOVA. A p-value of less than 0.05 was considered statistically significant. All values are presented as a group mean + standard error of mean (SEM).

## Results

### Metabolic activity of cells is donor-dependent only in flask based culture assays

Early passage MSCs were generated from 3 BM harvests from 3 different pigs. These cells were expanded in flasks to generate PreQE-P1, passage 2 and passage 3 cells (Fig. 1). Cells from each of these passages were subsequently evaluated for metabolic activity, morphological differences, growth kinetics, viability, surface marker expression, and functional potency.

**Fig. 3:**
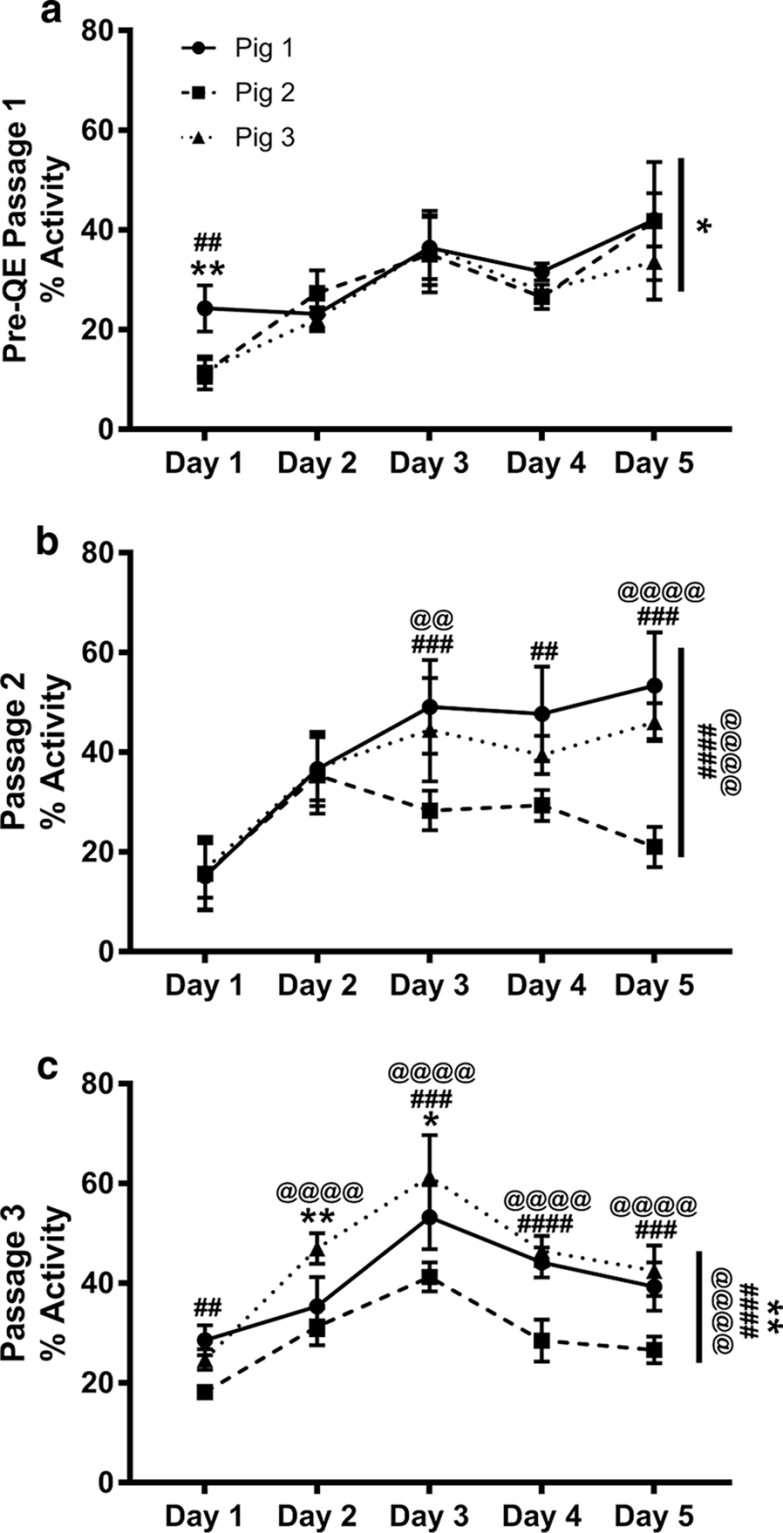
Donor and passage dependent variability in metabolic activity of MSC. Reduction of Alamar blue dye was assessed and compared between groups to determine metabolic activity of cells over a period of 1 week. **a** At PreQE-P1, group comparisons show differences between Pig 1 and 3 over the course of the week (indicated as overall significance on right side of the line graph). Differences on individual days as shown on graph. **b** At passage 2, group comparisons show differences between Pig 2 and Pigs 1 and 3 over the course of the week (indicated as overall significance on right side of the line graph). Differences on individual days as shown on graph. **c** At passage 3, group comparisons shows differences between Pigs 1, 2 and 3 over the course of the week (indicated as overall significance on right side of the line graph). Differences on individual days as shown on graph. Values are presented as mean ± SEM. These are calculated based on 4 wells/day/donor at each of the passages analyzed. ^#^Represents significant difference between Pigs 1 and 2, ^@^represents significant difference between Pigs 2 and 3, *represents significant difference between 1 and 3; *p < 0.05, **p < 0.01, ***p < 0.001, and ****p < 0.0001

First, we assessed metabolic activity of the cells by the Alamar Blue assay from each of the MSCs expanded in tissue culture flasks starting at PreQE-P1 and continuing to passages 2 and 3 in flasks (Fig. 1). Metabolically active cells reduce Alamar dye, which is evaluated by measuring absorbance. The amount of fluorescence is proportional to the number of living cells and corresponds to the cells’ metabolic activity. In contrast, nonviable cells with decreased metabolic activity generate lower signal as compared to healthy cells. Comparison of passage 1 cells from all three donors over 5 days is similar over time and shows no differences between donors (Fig. 3a), whereas, metabolic activity for cells from passages two and three are significantly different between donors (Fig. 3b, c). Cells from Pigs 1 and 3 have higher metabolic activity as compared to cells from Pig 2 over passages two and three.

### Metabolic activity is similar between cells expanded on quantum

MSCs selected from donor Pigs 1 and 3 were expanded in the Quantum^®^ Cell Expansion System from pre-Quantum expansion passage 1 through QE-1 and QE-2 (see “Materials and methods” and Fig. 2). Cells from Pig 2 were not expanded on quantum, due to poor yield in pre-quantum expansion passage 1. Media was sampled daily at nine different time points along the expansion process (pre-loading, days 1–8) for measurement of glucose consumption (Additional file 1: Figure S1A and C) and lactate production (Additional file 1: Figure S1B and D). These parameters serve as an approximation of metabolic activity and cellular proliferation rate. There were no significant differences found in either glucose consumption or lactate production between the donor pigs in either passage when expanded in the Quantum^®^ Cell Expansion System.

### MSCs from diverse donors exhibit distinct and persistent differences in cell morphology

**Fig. 4:**
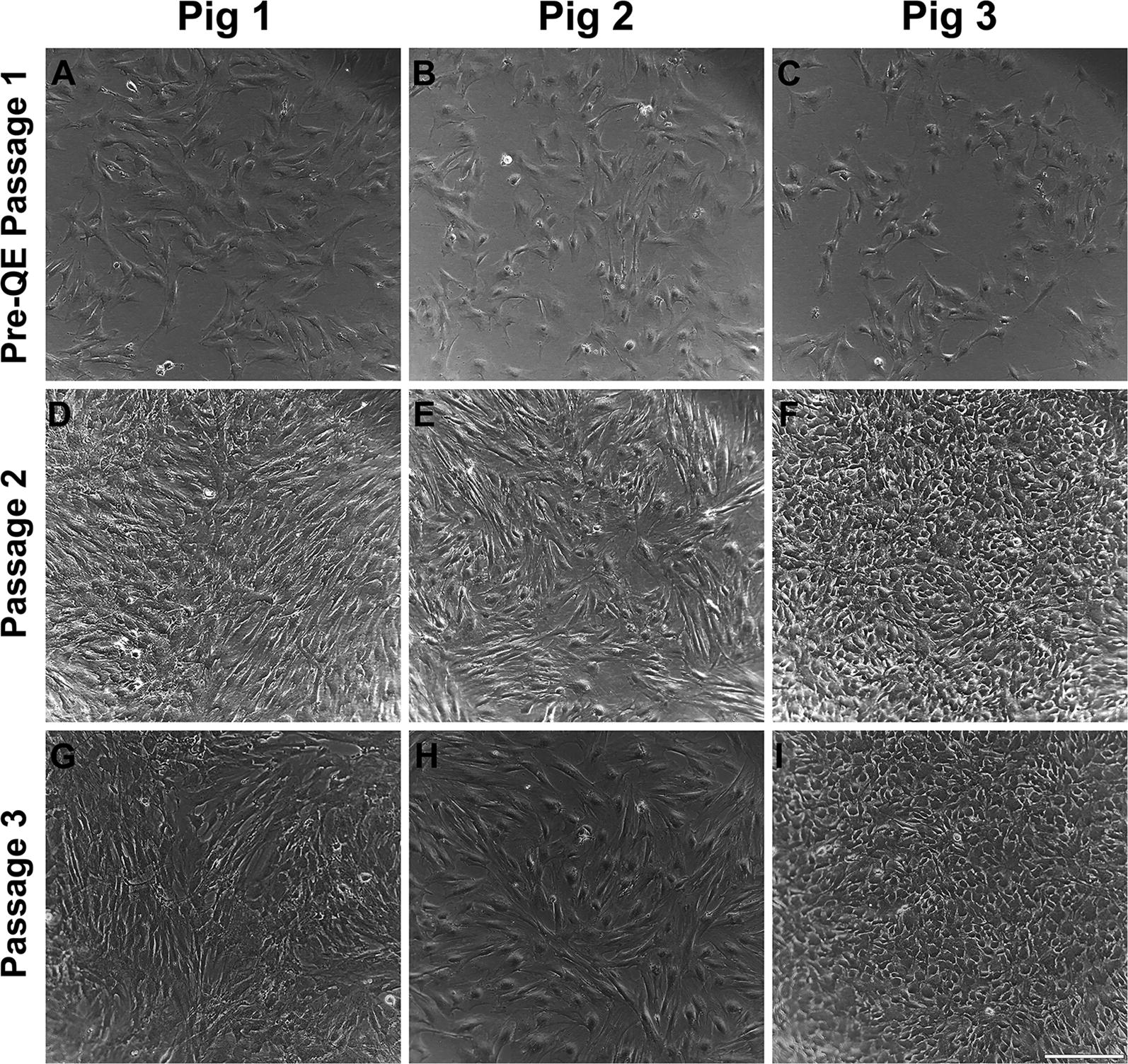
Morphological differences exhibited in MSCs between different donors. Bright field images of cells over a period of three passages were captured to highlight the morphology of cells over a time period. **a**–**i** Bright field images showing cultured swine MSCs from 3 different donor Pigs through 3 passages at 10× magnification. Rows indicate passage number. Columns indicate donor identity. Scale bar in panel I = 230 µm

To characterize cell morphology, MSCs from the three different donor pigs were cultured and observed through three passages. Pig 1 MSCs (Fig. 4a, d, g) displayed a characteristic fibroblast-like appearance with elongated, multipolar cell bodies, which became more spindle-shaped as cells neared confluence. Pig 2 MSCs (Fig. 4b, e, f) also displayed a fibroblastic appearance with elongated, multipolar cell bodies and adopted similarly spindle-shaped cell bodies as they neared confluence; however, many of the Pig 2 MSCs contained prominent and visibly larger nuclei as a distinguishing feature. Pig 3 MSCs (Fig. 4c, f, i) differed dramatically from both preceding pigs displaying an epithelial-like appearance that consisted of compact cell bodies, more regular dimensions and flattened cell bodies with polygonal shape. These cells adopted a cobblestone appearance as they neared confluence.

### Growth rate of cells is donor-dependent

**Fig. 5:**
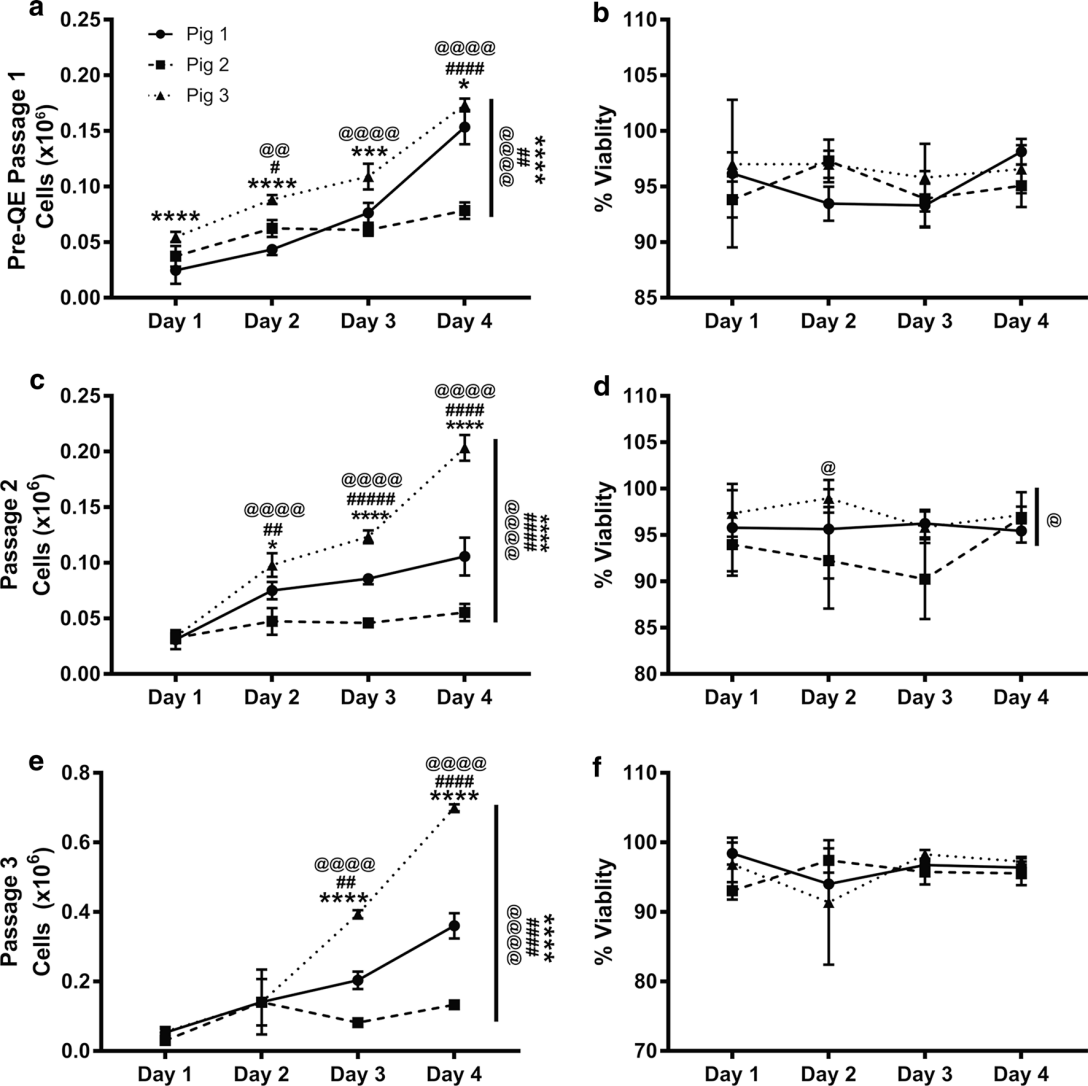
Cell growth rate is donor and passage dependent. **a** At PreQE-P1, group comparisons show differences between Pigs 1, 2 and 3 over the course of the week (indicated on right side of the line graph). Differences on individual days, as indicated on graph. **c** At passage 2, group comparisons showed differences between Pigs 1, 2, and 3 (indicated on right side of the line graph). Differences on individual days, as indicated on graph. **e** At passage 3, group comparisons showed differences for Pigs 1, 2 and 3 (indicated on right side of the line graph). Differences on individual days, as indicated on graph. **b**, **d**, **f** Among group comparisons and day to day comparisons at all 3 passages showed little to no differences in viability between donors. Values are presented as mean ± SEM. These are calculated from 3 wells/day/donor at each of the passages analyzed. ^#^Represents significant difference between Pigs 1 and 2, ^@^represents significant difference between Pigs 2 and 3, *represents significant difference between 1 and 3; *p < 0.05, **p < 0.01, ***p < 0.001, and ****p < 0.0001

To compare the growth rate of the three donors, we seeded the same number of cells in replicate wells and counted daily. Intergroup comparisons for each passage demonstrated that there were differences among each of the donors that become more distinct over the course of the three passages evaluated (Fig. 5a, c, e). Evaluation of the doubling time of each of the three donors (Table 1) revealed that cells derived from Pig 3 and Pig 1 went through approximately 2 population doublings over a course of 1 week (for both the passages evaluated), however cells from Pig 2 went through less than 1 population doubling over the same period of time. The fold increase in cell number followed a similar pattern. Despite the differences in cell counts and population doubling times, there were no differences in viability among donors across each passage (Fig. 5b, d, f).

**Table 1:** Cell growth and population increase over the course of each passage

Donor ID	Population doubling	Fold increase
PreQE-P1 → passage 2		
Pig 1	1.77	3.40
Pig 2	0.59	1.51
Pig 3	2.15	4.42
Passage 2 → passage 3		
Pig 1	2.18	4.52
Pig 2	0.76	1.69
Pig 3	2.3	4.93

Over a week, population doubled *n* times and population increased by *n* fold

### MSC from different donors express comparable cell surface markers

**Fig. 6:**
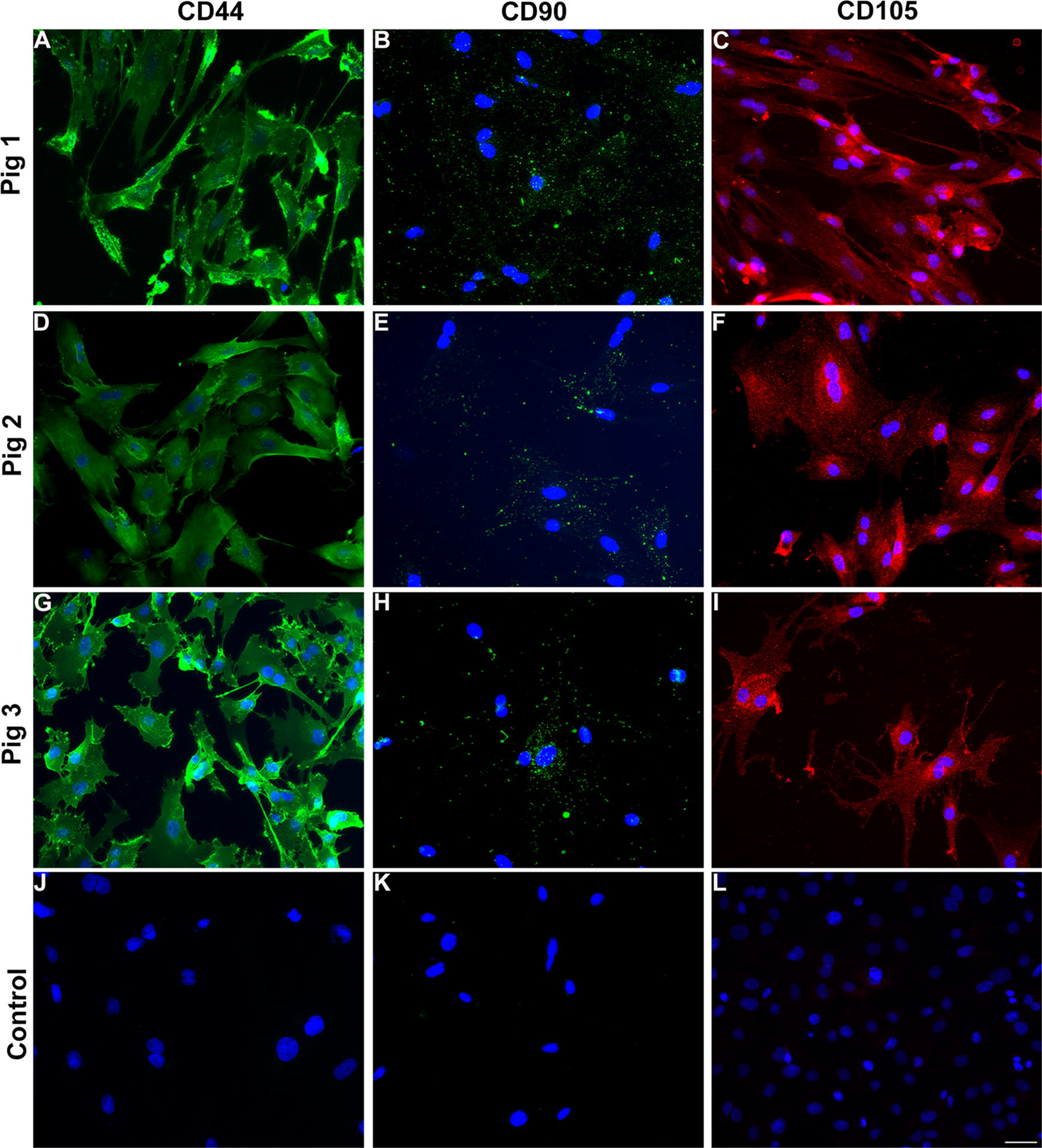
Immunophenotyping reveals expression of markers by all three lines of donor derived MSC at passage 3. Cells grown in culture were stained with CD44 (**a**, **d**, **j**), CD90 (**b**, **e**, **h**) or CD105 (**c**, **f**, **i**) and nuclei were counterstained with DAPI. Control panels indicate secondary antibody incubation alone. Scale bar in **l** is 100 μm

MSCs are characterized based on their ability to adhere to plastic, expression of cell surface markers such as CD44, CD105 and CD90 and ability to differentiate into osteoblasts, adipocytes and chondrocytes. Here we examine the expression of these MSC cell markers from all three donors at passage 3 in culture by immunocytochemistry (Fig. 6). CD44, is a protein that binds to hyaluronan and is involved in cell growth, and migration. CD90, is a glycoprotein known to be expressed by MSC extracellular vesicles. CD105 is a transmembrane receptor for TGF-beta superfamily ligands. Similar expression patterns of CD90 and CD105 were observed between the cells from different donors, but CD44 was expressed at lower levels (based on overall signal intensity) from donor Pig 2 as compared to the other two donors. These data are consistent with the slower growth rate observed for cells from donor Pig 2.

**Fig. 7:**
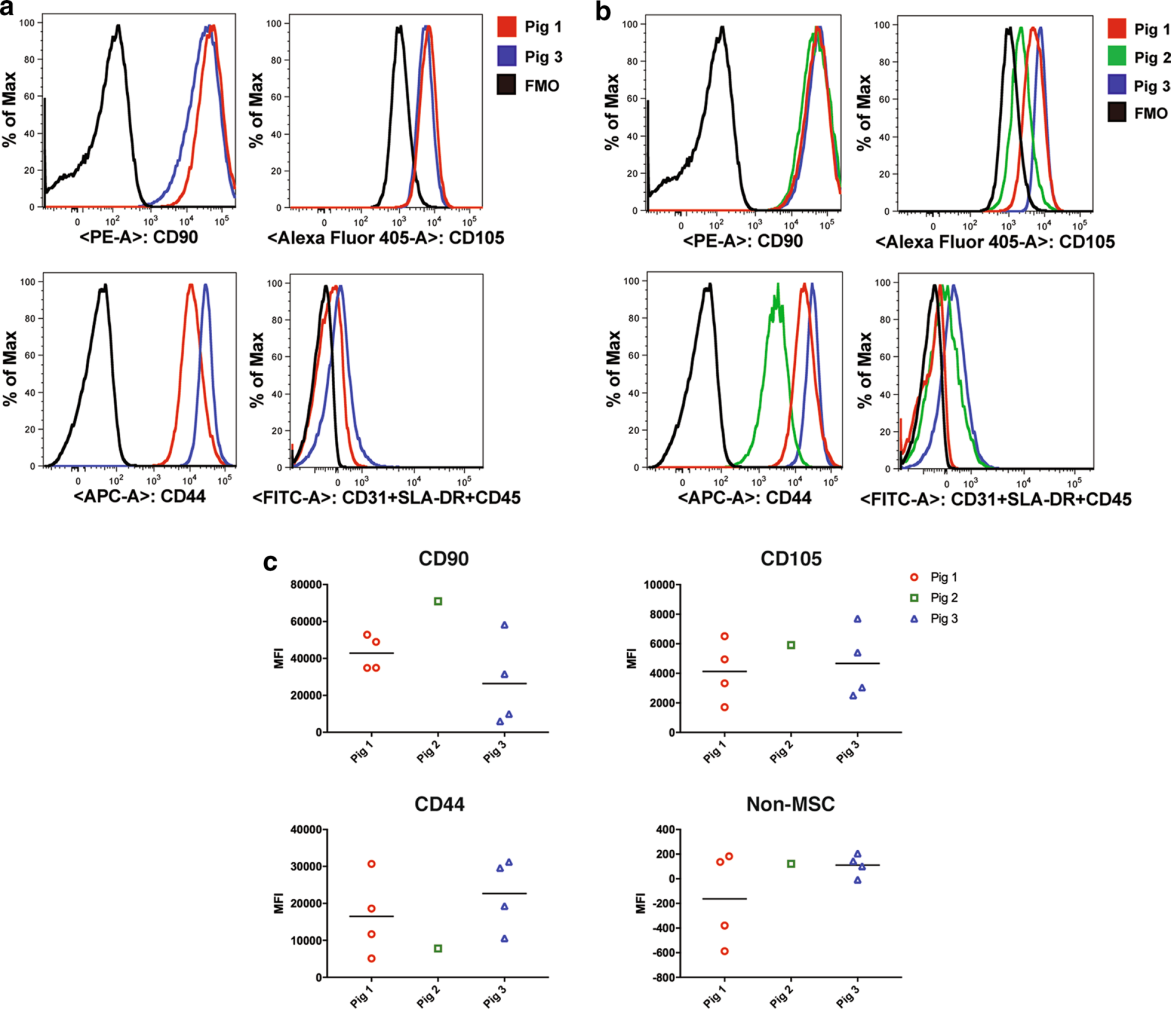
Frequency of MSC markers is maintained on QE1, QE2 and manually expanded passage 3 cells. Cryopreserved MSCs from donor Pigs 1, 2, and 3 were thawed and stained with mesenchymal stem cell markers CD44-APC, CD90-PE, and CD105-Alexa405 along with a FITC cocktail containing lineage markers CD45, CD31, and SLA-DR Class II. Cells were pregated to exclude debris and doublets before gating on total live cells. **a** Frequency of CD90, CD44, and CD105 positive cells within QE1 cells of each donor Pig. **b** Frequency of CD90, CD44, and CD105 positive cells within QE2 cells for Pigs 1 and 3 and manually expanded Passage 3 cells of Pig 2 grown in tissue culture flasks. **c** Quantitative assessment of CD90, CD44, and CD105 positive cells within QE2 cells for Pigs 1 and 3 and manually expanded passage 3 cells of Pig 2 grown in tissue culture flasks

The expression of surface markers was confirmed and quantitated by flow cytometry, which demonstrated that the majority of QE-1 (Fig. 7a), QE-2 and passage 3 cells (Fig. 7b, c) from all donor pigs express MSC markers CD90, CD44, and CD105 and are negative for non-MSC markers CD31, SLA-DR, CD45+ (Fig. 7). Due to restricted expansion and limited cells numbers at PreQE-P1, immunophenotyping of Pig 2 cells was from manually expanded and not quantum expansion as for cells from Pigs 1 and 3. Thus, additional rounds of expansion on quantum did not alter level of MSC cell marker expression. There was no significant difference in MSC marker expression among Pigs for CD90 or CD105 (Fig. 7b, c), nonetheless, cells from Pig 2 expressed low levels of CD44 as compared to cells from other two pigs. This data is consistent with our immunocytochemistry data.

### MSCs from Pig 1 and Pig 3 display tri-lineage differentiation potential

**Fig. 8:**
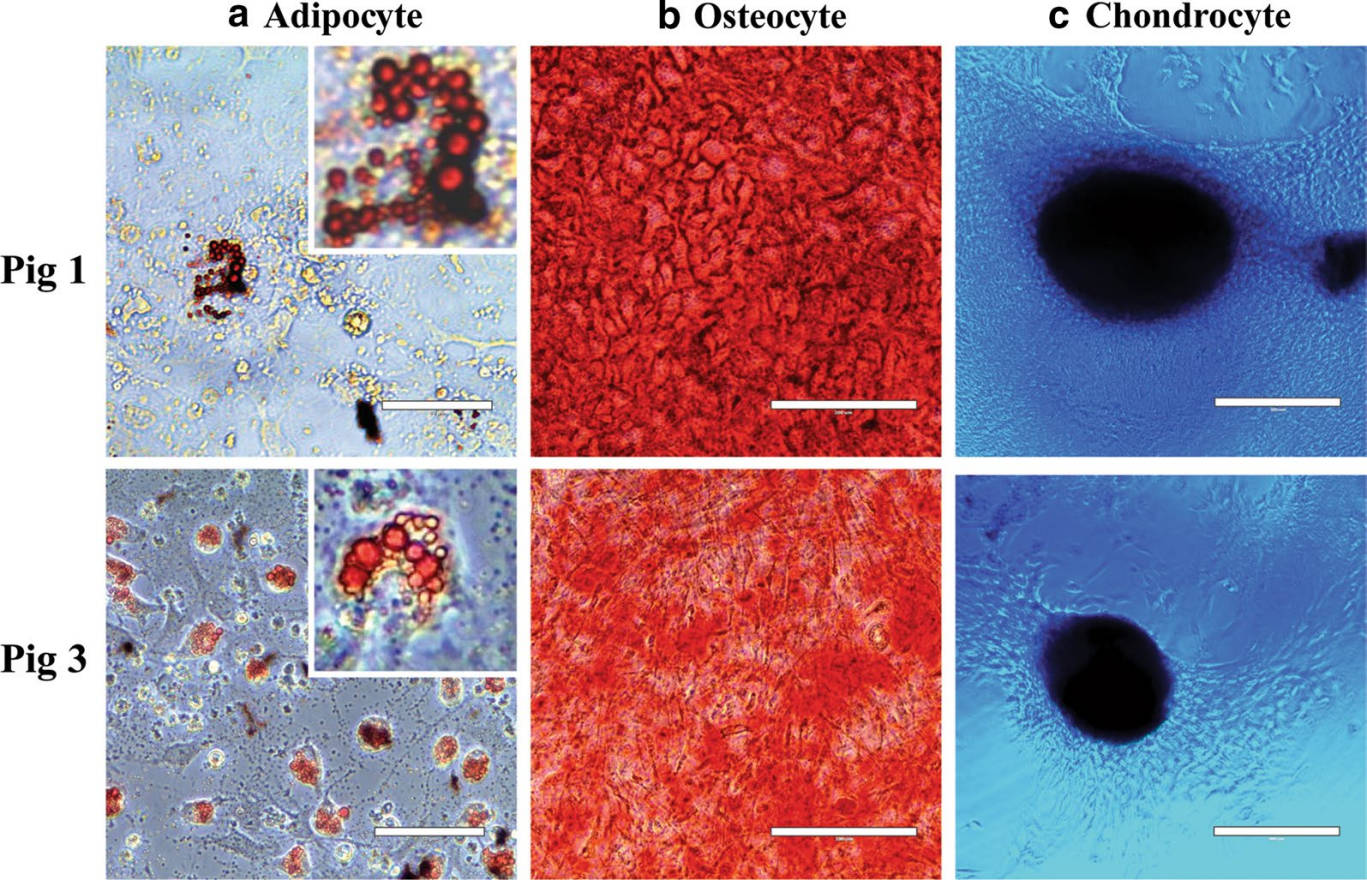
QE2 expanded MSCs are capable of multilineage differentiation. **a** Oil Red O staining of adipocytes grown for 5–7 days, scale bars represent 50 µm. **b** Alizarin Red S staining of osteocytes grown for 14 days, scale bars represent 200 µm. **c** Alcian Blue staining of chondrocytes spheroids grown for 14 days. Scale bars represent 400 µm. All images were captured under bright field using a light microscope. Photos are representative of replicate experiments done in triplicate

When subjected to the appropriate media for adipocyte, osteocyte, and chondrocyte differentiation, QE-2 cells from Pig 1 and Pig 3 both demonstrated differentiation capacity by positive staining for each lineage (Fig. 8). Cells from Pig 2 did not proliferate and expand enough to be assayed for cell differentiation. When comparing each lineage stain, Pig 3 appears to have produced more adipocytes, as indicated by more cells with intact lipid structures staining positive for Oil Red O compared to Pig 1 (Fig. 8a). Pig 1 adipocytes, while less abundant, have larger lipid vacuoles and are more cytoplasmic when compared to those of Pig 3. Alternatively, osteocyte growth appears higher in Pig 1, as demonstrated by more cells taking up the alizarin red staining for calcium deposits (Fig. 8b). Pig 1 MSCs also appear to differentiate into osteocytes at a faster rate than Pig 3 cells, which take up the alizarin red stain at an earlier time point in culture when both are grown in parallel. Both pigs had comparable Alcian Blue staining of chondrocyte spheroids, with Pig 1 developing a larger spheroid than Pig 3 (Fig. 8c).

### Functional potency of MSC conditioned media on endothelial monolayer permeability

**Fig. 9:**
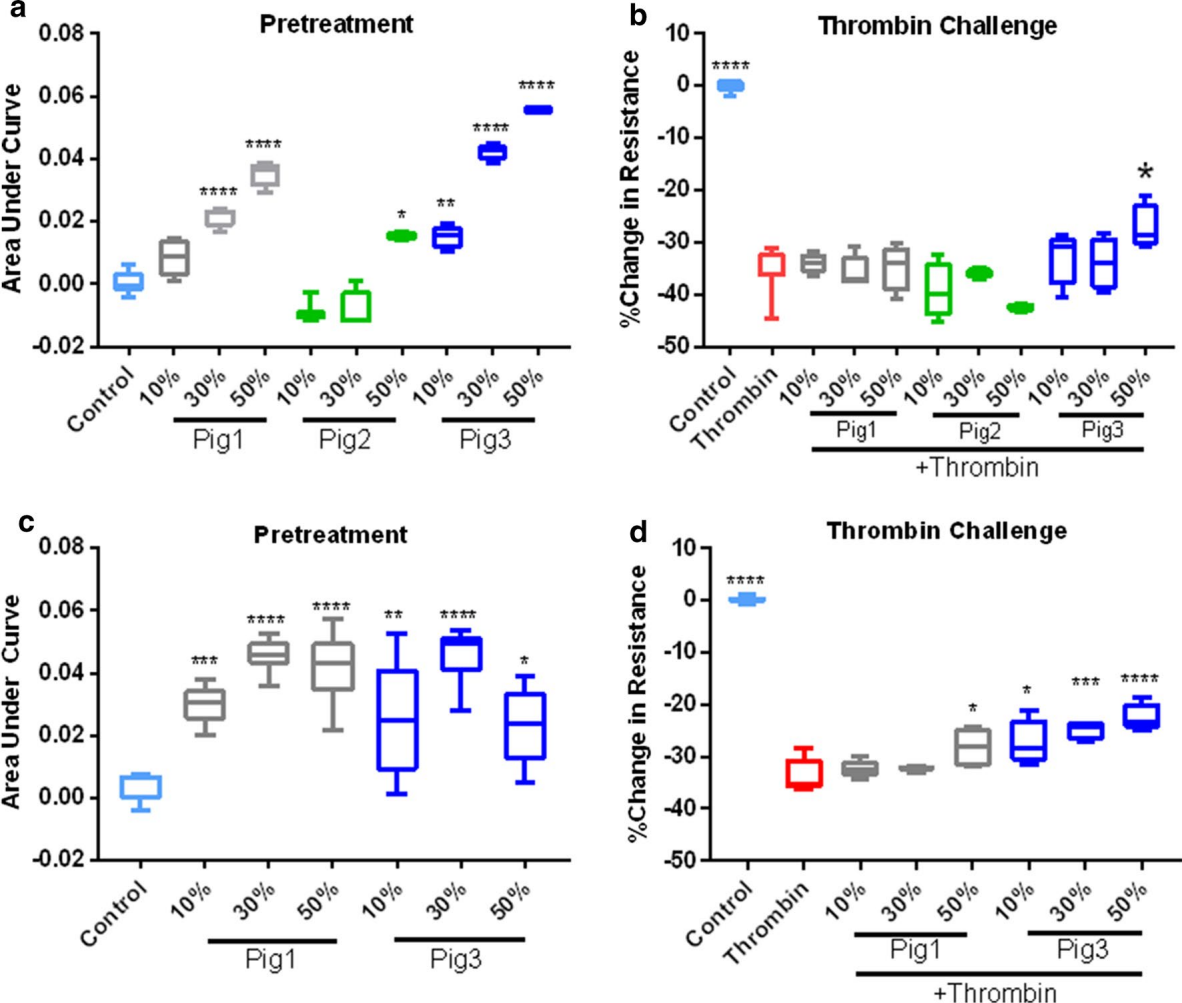
Effect of MSC on endothelial barrier function. Monolayer transendothelial electrical resistance (TEER) of HPMVEC cells treated with control MSC media, 10% conditioned media (CM), 30% CM, or 50% CM, generated from manually expanded Pig 1, Pig 2, or Pig 3 cells (**a**), and their effect against thrombin challenge (**b**). The effect of CM generated from QE-2 Pig 1 and Pig 3 cells on HMVEC barrier resistance (**c**), and their effect against thrombin challenge (**d**). AUC box plots represent area under the curve quantitation of barrier resistance as mean ± STD. % Change in resistance boxplots represent a maximum decrease in barrier resistance as mean ± STD. These are calculated based on 4 wells/condition/donor/treatment (dose). *Indicates significant difference from control in **a**, **c**, while * indicates a significant difference from thrombin in **b**, **d**, as determined by one-way ANOVA (*p < 0.05, **p < 0.01, ***p < 0.001, ****p < 0.0001)

Utilizing the ECIS system to measure the transendothelial electrical resistance (TEER) of the endothelial barrier junctions, we compared the effects of conditioned media (CM) generated from MSCs on the integrity of pulmonary endothelial cell (HPMVEC) monolayers. Treatment with CM from both manually expanded cells, and QE-2 cells, induced an increase in endothelial barrier resistance, in a dose dependent manner (Fig. 9a, c). For manually expanded, passage 3 cells from Pig 1, whereas, 10% CM was not statistically different as compared to control, both 30% CM and 50% CM pretreatment groups resulted in an increase in resistance. For Pig 2 generated CM no statistically significant differences were observed at lower concentrations as compared to control, but the 50% CM dose did increase resistance. In contrast, Pig 3 generated CM was protective (increased resistance as compared to control) at three concentrations tested (Fig. 9a, Additional file 2: Figure S2). Taken together, the highest dose (50%) of CM generated from each donor appeared to tighten endothelial barrier resistance. We next compared, CM from QE-2 generated cells and similar effects were observed for Pigs 1 and 3. CM media form both pigs were protective at all concentrations tested, as seen by increased resistance in all treatment groups as compared to control (Fig. 9c, Additional file 3: Figure S3). Altogether, the results suggests both the manually expanded and QE-2 cells stimulate an increase in endothelial barrier resistance.

We further examined the effect of MSC-CM from different donors on thrombin challenged monolayers. Thrombin, a known inducer of barrier disruption, resulted in a decrease in resistance of 36% and 34% (Fig. 9b, d). For manually expanded cells from passage 3, pretreatment with CM at all concentrations did not significantly attenuate thrombin induced disruption, except for CM from Pig 3, where 50% CM pretreatment resulted in a thrombin induced decrease of 29% when compared to control, a significant protection of barrier as compared to the thrombin group (p < 0.05) (Fig. 9b, Additional file 2: Figure S2). In comparison, CM generated from QE-2 cells exhibited enhanced barrier protection against thrombin (Fig. 9d). The 50% dose of Pig 1 CM pretreatment resulted in a decrease in resistance of 28%, when compared to control, a significant attenuation of barrier disruption as compared to the thrombin group (p < 0.05). Pretreatment of all doses (10%, 30%, 50%) of Pig 3 CM resulted in a significant reduction of thrombin induced barrier disruption; 27% (p < 0.05), 19% (p < 0.001), 22% (p < 0.0001), decrease in resistance, respectively (Fig. 9d, Additional file 3: Figure S3). Taken together, in both manual expansion (passage 3), and QE-2 expansion, CM from Pig 3 cells appeared to attenuate the effects of thrombin challenge on the endothelial monolayer. Only QE-2 Pig 1 CM seemed to protect against thrombin challenge. CM from Pig 2 appeared to have no effect on the thrombin treated monolayer.

## Discussion

In this study, we sought to characterize swine BM-MSCs with the ultimate goal of selecting optimal cells based on growth characteristics, marker expression, differentiation potential and potency, for large scale expansion and testing in a swine model of trauma induced ARDS. In the area of MSC clinical or pre-clinical dose production, one of the major challenges in the field is producing enough cells with the desired biological effect. This paper focuses on characterization of MSCs derived from three different donor bone marrows for large scale expansion. Three independent swine donor derived MSCs were compared for their growth kinetics, metabolic activity, morphological differences, viability, surface marker expression, functional potency and ability to differentiate into three known lineages of MSC differentiation: adipocytes, osteocytes, and chondrocytes. Although the cells from individual donors displayed similar characteristics in MSC surface marker expression and differentiation capacity, we found variability in growth potential, doubling time, metabolic activity and more importantly a functional measure of endothelial cell barrier permeability.

For functional assessment of the cell potency, endothelial cell (EC) permeability was measured. MSCs derived from one of the donors, Pig 2, displayed stunted expansion capabilities with increasing passage and conditioned media derived from these cells were not as potent in inhibiting endothelial cell permeability as the other two cells tested. Studies with human MSCs have shown that senescent cells are compromised in their survival ability, functionality, and their capability to curb inflammation [4], which is a similar phenotype to what we observed with Pig 2 MSCs. Endothelial permeability as measure of potency is relevant to trauma induced ARDS, since pulmonary vascular permeability and pulmonary edema is a key feature of clinical ARDS [10, 11]. Vascular permeability is also relevant in other forms of injury such as traumatic brain injury (TBI), where the blood brain barrier is compromised, leading to vascular leak, cerebral edema and mortality in severe cases. Our previous studies have demonstrated that MSCs attenuate injury induced vascular leakage in two different pre-clinical murine trauma models, TBI and hemorrhagic shock (HS) induced ARDS [12–14]. Particularly in a rat and a mouse model of hemorrhagic shock, we examined the disruption of vascular permeability in lungs [14, 15]. Both MSCs and MSC extracellular vesicles significantly attenuate the permeability to a 10 kDa dextran after controlled hemorrhage in mice [14, 15]. Hence, our choice of EC permeability as a functional measure of cell potency is relevant to the swine trauma model in which we intend to test the expanded MSCs. In this paper, we demonstrate functional differences on endothelial permeability between donors when evaluating the potency of the MSC conditioned media (CM), which contains EVs and soluble potency factors [16]. Pig 1 and Pig 3 MSC CM displayed protection of EC monolayer permeability, whereas Pig 2 displayed no protection at lower doses tested, suggesting that there are cell dependent differences in MSC functional potency. Interestingly, after challenge with thrombin, a known inducer of EC permeability, we found that only Pig 3 (expanded manually) at the highest dose of CM was able to offer protection. Whereas CM derived from QE-2 cells were protective at all concentrations tested for Pig 3 but only at the highest concentration for Pig 1.

Our studies demonstrate that MSCs from all three donors express similar levels of characteristic MSC surface markers. Histological analysis of cells show that MSCs derived from all three donors express MSC markers. It was observed that signal intensity for CD44 was lower for cells derived from Pig 2 as compared to cells derived from the other two donors. This finding was confirmed by quantitative flow cytometry analysis that showed decreased expression of CD44 in cells derived from Pig 2. CD44 is a cell surface marker that interacts with multiple ligands of the extracellular matrix, such as hyaluronan, selectins, collagen, and fibronectin. CD44 is involved in various cellular functions including cell proliferation, differentiation, migration, presentation of cytokines and chemokines, and signaling for cell survival. Decreased expression of this receptor is consistent with poor growth and survival observed for cells derived from Pig 2.

In our study, the test of functional potency, inhibition of endothelial permeability, demonstrates significant differences between the cells. Our studies demonstrate that even though cells from Pig 2 express all MSC cell surface markers they are not equally potent in protecting the endothelial barrier. Taken together, our findings on cell growth, metabolism, and potency suggest that Pig 3 is the donor of choice for dose production to be tested in the pre-clinical swine model of polytrauma ARDS. These findings suggest that while screening MSCs an application relevant potency assay should be included in addition to marker expression and differentiation potential tests. There should be a few assays other than marker expression that can be standardized as minimally required for screening, as is currently done for any blood products. For developing cellular therapeutics, other than growth characteristics, it is pertinent that potency assays relevant to the clinical application be used for screening donors.

Recent pre-clinical studies have highlighted the necessity to characterize the MSCs for functional properties and potency not only by the donor from which they are obtained, but also by the tissue they are derived from. MSCs can be isolated from multiple tissue sources such as adipose tissue, umbilical cord, bone marrow and placenta [17]. These different sources of MSCs are demonstrated to have differing secretomes and different capacities to differentiate into particular cell types [18]. MSCs derived from adipose tissue and BM differed in their secretion of factors involved in neurogenesis, oxidative stress and excitotoxicity [19]. It is important to note that there is considerable inter-donor variability in MSCs as well [20]. In a recent study, expression of prostaglandin E2 (PGE_2_) by MSCs is shown to correlate with potency of the MSCs in pre-clinical models of TBI [21, 22]. It is likely that in the future subpopulations of cells may be sorted from a particular donor for expansion and dose production depending on the specific clinical application of the cells.

This study highlights the need to determine the growth characteristics and application dependent potency of cells collected from multiple donors based on in vitro and pre-clinical model testing before they are selected for large scale expansion and clinical dose production [22]. An approach of screening cells that is application and potency dependent, may be a future direction in MSC therapeutics.

## Conclusions

The qualities of MSCs such as their ability to differentiate into multiple lineages, migrate towards site of injury, immunomodulation and their capacity to secrete factors have made them an attractive cell based therapy in tissue repair and multiple diseases. While MSC therapy is very promising, the lack of unique phenotypic markers, and non-standardized extraction and expansion methodologies are currently limiting their use. To generate pre-clinical and clinical MSC dose production, we need to establish minimum standards for quality control. Our study highlights that for production of MSCs for cell-based therapy, it is imperative to examine donor and derived cell characteristics for not only marker expression, growth and differentiation characteristics but also to test potency in application dependent assays prior to selection of the optimal cell lineage for large scale expansion and dose production.

## Additional files

**Additional file 1: Figure S1.** Metabolic activity of MSCs by donor when expanded on Quantum. (A-D) MSCs from Pig 1 and Pig 3 were cultured in Quantum^®^ Cell Expansion System (QE-1—A, B; QE-2—C, D). Media was sampled daily from pre-loading stage through harvest day for measurement of glucose consumption (A, C) and lactate production (B, D) throughout cell expansion phase to approximate cell growth and metabolism.

**Additional file 2: Figure S2.** Effect of manually expanded MSC-CM on thrombin mediated endothelial barrier disruption. TEER ECIS tracing of human pulmonary microvascular endothelial cells pretreated with a dose curve of conditioned media (10%, 30%, 50%) generated from Pig 1 (A), Pig 2 (B), or Pig 3 (C), and subsequently challenged with thrombin (0.2 U/ml) (D–F).

**Additional file 3: Figure S3.** Effect of QE-2 expanded MSC-CM on thrombin mediated endothelial barrier disruption. TEER ECIS tracing of human pulmonary microvascular endothelial cells pretreated with a dose curve of conditioned media (10%, 30%, 50%) generated from Pig 1 (A) or Pig 3 (B), and subsequently challenged with thrombin (0.2 U/ml) (C and D).

## Abbreviations

BM-MSCs: bone marrow derived mesenchymal stem cells (also known as mesenchymal stromal cells)
BM: bone marrow
EVs: extracellular vesicles
ARDS: acute respiratory distress syndrome
PaO2: partial pressure of arterial oxygen
FiO2: percentage of inspired oxygen
IACUC: Institutional Animal Care and Use Committee
ACURO: The Department of Defense Animal Care and Use Review Office
MEM: minimal essential medium
FBS: fetal bovine serum
PBS: phosphate buffered saline
HPMVEC: human pulmonary microvascular endothelial cells
ECIS: electric cell-substrate impedance sensing system
TEER: transendothelial electrical resistance
AUC: area under curve
CM: conditioned media
EC: endothelial cell
TBI: traumatic brain injury
HS: hemorrhagic shock
PGE_2_: prostaglandin E2
