# Supplementary figures and images for: Bone marrow donor selection and characterization of MSCs is critical for pre-clinical and clinical cell dose production
Source: J Transl Med. 2019 Apr 17;17:128. doi: 10.1186/s12967-019-1877-4 (PMC6469059; doi:10.1186/s12967-019-1877-4)

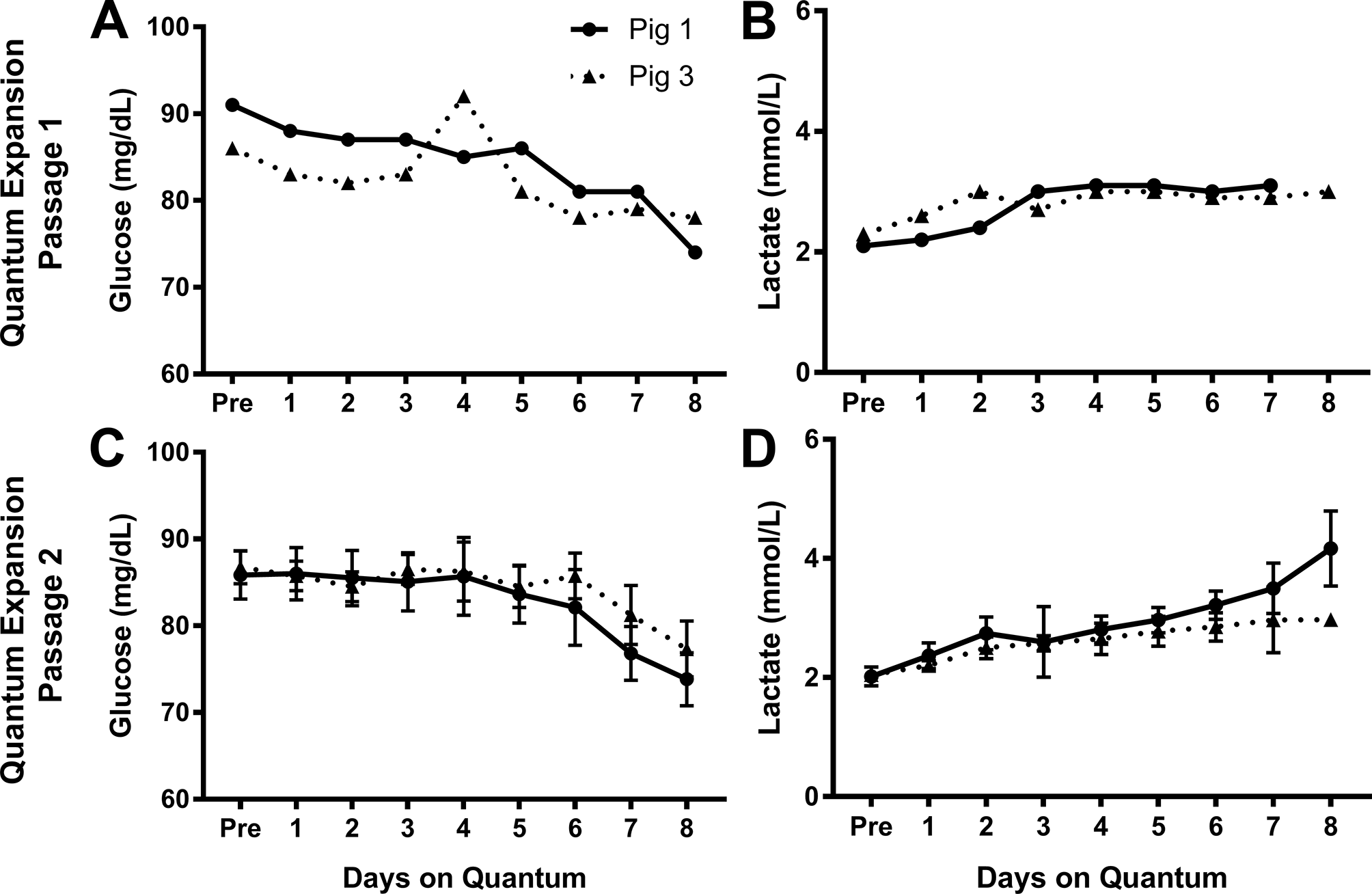

Supplement: Supplementary file 1 — Additional file 1: Figure S1. Metabolic activity of MSCs by donor when expanded on Quantum. (A-D) MSCs from Pig 1 and Pig 3 were cultured in Quantum® Cell Expansion System (QE-1—A, B; QE-2—C, D). Media was sampled daily from pre-loading stage through harvest day for measurement of glucose consumption (A, C) and lactate production (B, D) throughout cell expansion phase to approximate cell growth and metabolism. [file 12967_2019_1877_MOESM1_ESM.tif]

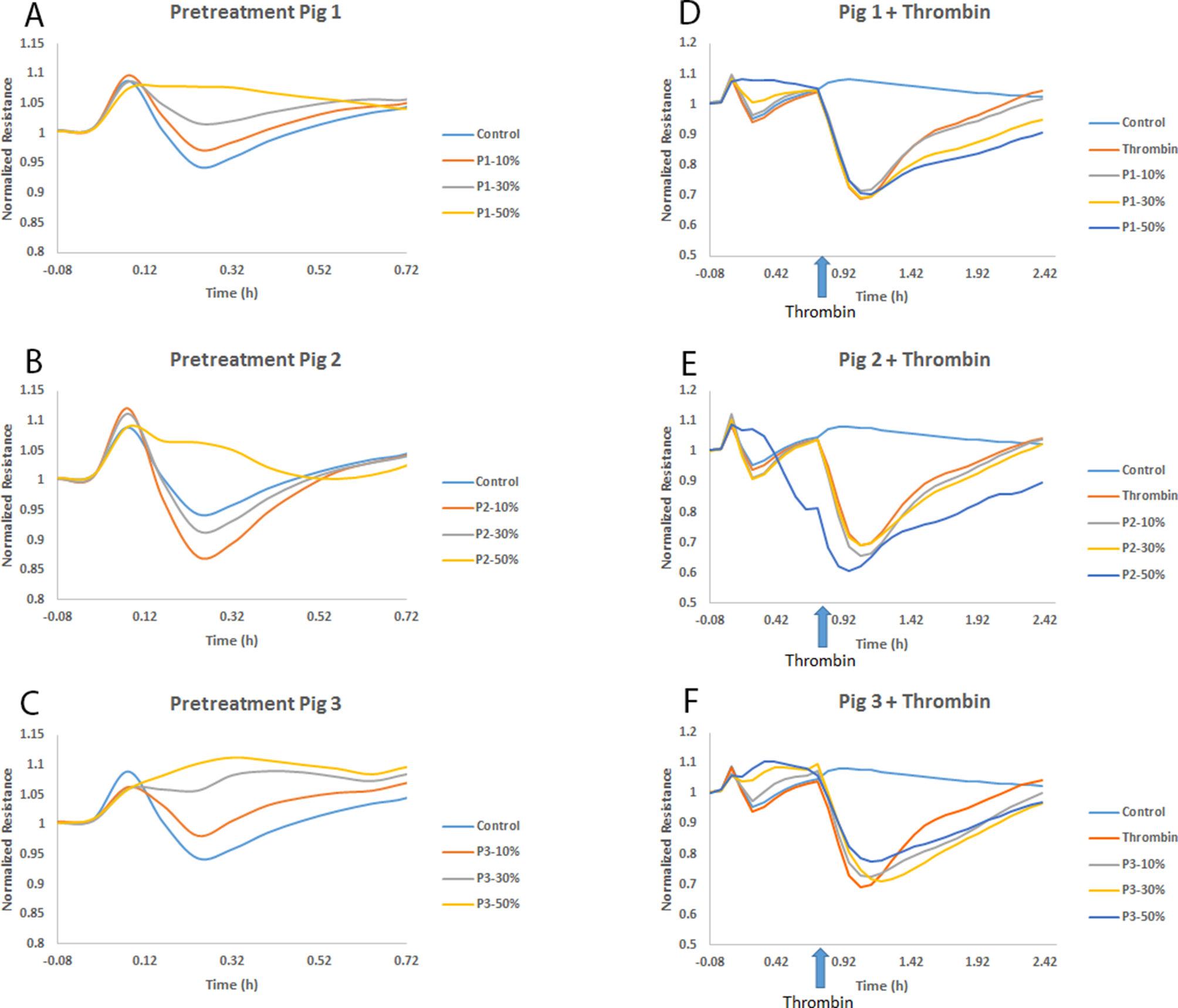

Supplement: Supplementary file 2 — Additional file 2: Figure S2. Effect of manually expanded MSC-CM on thrombin mediated endothelial barrier disruption. TEER ECIS tracing of human pulmonary microvascular endothelial cells pretreated with a dose curve of conditioned media (10%, 30%, 50%) generated from Pig 1 (A), Pig 2 (B), or Pig 3 (C), and subsequently challenged with thrombin (0.2 U/ml) (D–F). [file 12967_2019_1877_MOESM2_ESM.tif]

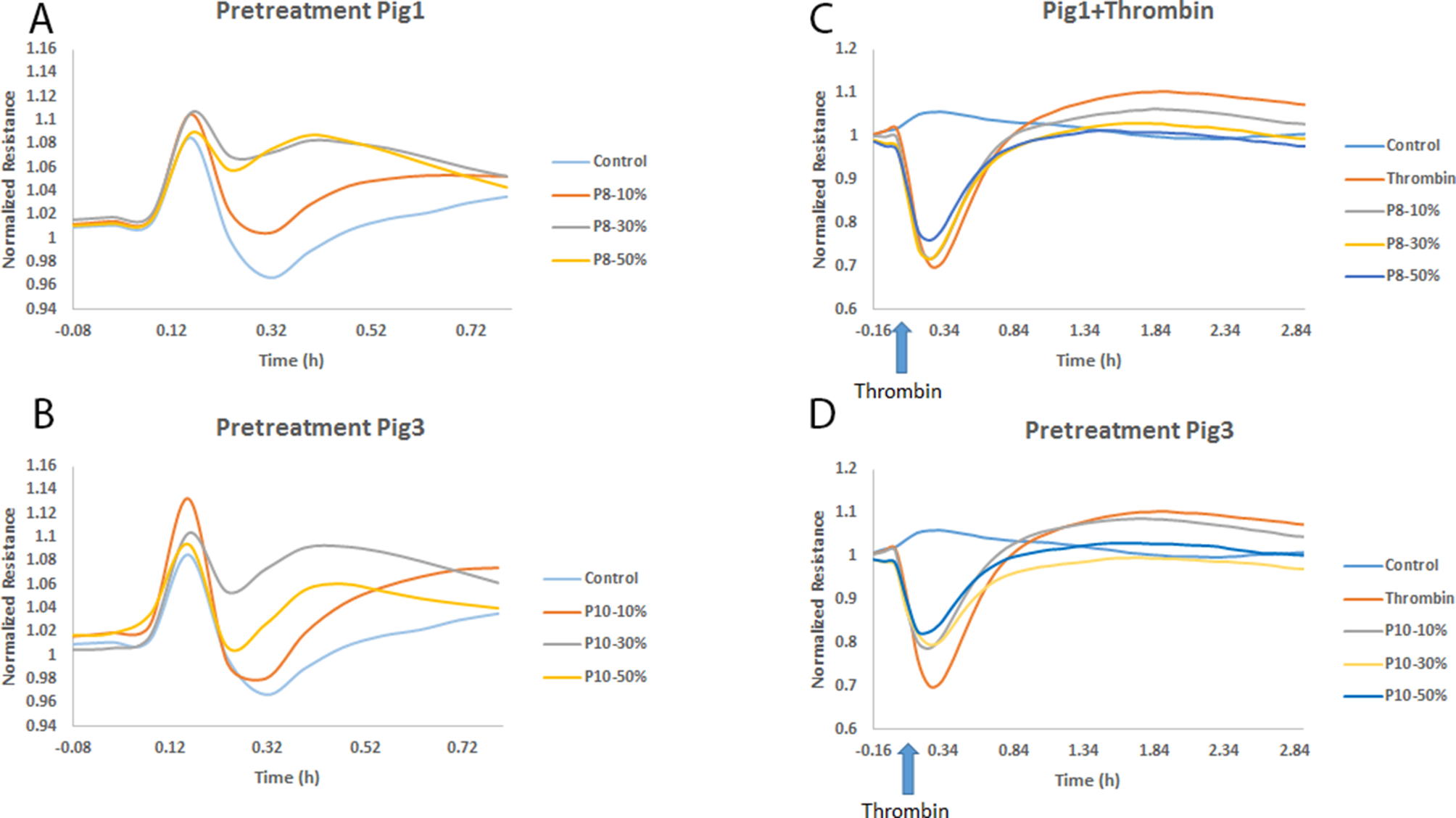

Supplement: Supplementary file 3 — Additional file 3: Figure S3. Effect of QE-2 expanded MSC-CM on thrombin mediated endothelial barrier disruption. TEER ECIS tracing of human pulmonary microvascular endothelial cells pretreated with a dose curve of conditioned media (10%, 30%, 50%) generated from Pig 1 (A) or Pig 3 (B), and subsequently challenged with thrombin (0.2 U/ml) (C and D). [file 12967_2019_1877_MOESM3_ESM.tif]
